# Qualitative perspectives toward prostitution's perceived lifestyle addictiveness

**DOI:** 10.1556/JBA.2.2013.013

**Published:** 2013-10-15

**Authors:** Michael W. Firmin, Alisha D. Lee, Ruth L. Firmin, Lauren Mccotter Deakin, Hannah J. Holmes

**Affiliations:** ^1^Cedarville University, Cedarville, OH, USA *; ^2^University of Toledo, Toledo, OH, USA; ^3^Indiana University – Purdue University at Indianapolis, Indianapolis, IN, USA; ^4^Richmont Graduate University, Atlanta, GA, USA; ^5^Wayne State University, Detroit, MI, USA

**Keywords:** prostitution, sex work, qualitative research, psychological addictions

## Abstract

*Background and aims:* The aim of the present study was to provide a phenomenological perspective of individuals who actively engage in street-level prostitution and identified a lifestyle addiction associated with their activities. *Methods:* We interviewed 25 women who were incarcerated in American county jails (at the time of interviews) for prostitution crimes. The transcripts were analyzed for themes that represented the shared consensus of the research participants. *Results:* Four negative psychological dynamics related to prostitution. First, participants described accounts of physical and emotional violence which they experienced at the hand of clients and others involved in the lifestyle. Second, interviewees explained an extreme dislike for their actions relating to and involving prostitution. These individuals did not describe themselves as being sexually addicted; sex was means to a desired end. Third, participants described how prostitution's lifestyle had evolved into something which they conceptualized as an addiction. As such, they did not describe themselves as feeling addicted to sex acts – but to lifestyle elements that accompanied prostitution behaviors. Finally, participants believed that freedom from prostitution's lifestyle would require social service assistance in order to overcome their lifestyle addiction. *Conclusions:* The results show that, although the prostitutes repeatedly and consistently used the term “addiction” when describing their lifestyles, they did not meet the DSM-IV-TR criteria for addiction. Rather, they shared many of the same psychological constructs as do addicts (e.g., feeling trapped, desiring escape, needing help to change), but they did not meet medical criteria for addictive dependence (e.g., tolerance or withdrawal).

Spice's (2007) definition of prostitution emphasizes the wide range of backgrounds from which women are lead into illicit sex work, including escort services, brothels, and street-level work. Spice further notes that prostitutes generally hold common values which motivate and often drive their behavior, despite the diversity of ethnicities, personal histories, education levels, and life experiences of these women. While it is hard to estimate the exact number of women working as prostitutes in the United States, the National Task Force on Prostitution (2008) estimated the number to be approximately two million. This staggering level of participation makes research regarding prostitution – including motivations for entry into and continuance in prostitution, effects of prostitution, and best practices for assisting women currently or previously involved with prostitution – a critical matter.

Main (2012) reported that those women who engage in “street-level” contexts of prostitution, one of the most common forms of sex work, have often received the lowest pay. Furthermore, Reid (2011) and Miller (1993) found that women who were “street-prostitutes” encountered higher instances of abuse and violence than did those women who engaged in other contexts of sex work. In light of Miller's (1993) findings, strong underlying motivations seemingly are held by most women who regularly engage in sex work – and particularly street-level prostitution – considering they risk personal health and safety in order to continue in their prostitution behaviors.

While our study focuses on the detrimental psychological dynamics involved with dynamics involved with prostitution, other researchers have explored the experiences and specific actions taken by women engaged in the behaviors. For example, common experiences include health risks and physical harm (Raymond, Hughes & Gomez, 2010). Sanders (2004) reported that sex workers often feel ready to cope with health risks such as sexually transmitted diseases, seeing them as simply “part of the job”. Additionally, sex workers often more readily recognize the risk of physical harm, which Spice (2007) labels the greatest threat to the well-being of women who engage in prostitution. In addition, past researchers also have investigated steps which women have taken in order to protect their physical safety. Both O'Doherty (2011) and Williamson and Folaron (2003) reported that women tend to rely on their intuition when determining the likelihood that a client may hurt them, making exchanges in visible areas, and sometimes carrying a small weapon in order to protect themselves from physical harm. The central focus of most previous research has been on the Potential physical harm that may come to women involved in prostitution and the ways in which the women can maintain physical safety (Heilemann & Santhiveeran, 2011).

Much less research has evaluated the negative impact that sex work may have on the psychological health of these women. Furthermore, it is much more difficult to take practical steps to protect one's psychological health than it is to protect one's physical health. For this reason, we believe it beneficial to investigate the psychological dynamics, including strain, that women experience as a result of prostitution.

Despite the obvious existence of multiple risks that accompany involvement in street-level prostitution, Lucas (2005) reported the women in her sample insisted that their involvement in prostitution was the result of their own, personal choices. Furthermore, the participants seemingly perceived prostitution as something that enhanced their ability to adapt to certain situations outside of the prostitution environment. Belcher and Herr (2005) advance the findings in that women engaged in prostitution often were focused on temporary, short-term rewards. Overall, participants placed high value on more immediate-gratification motivations, such as money. While experiencing some perceived short-term perceived benefit, the women admittedly allowed themselves to be controlled by extrinsic, immediate gratification factors, leaving them feeling hopeless and unable to exit the lifestyle. These results are congruent with Williamson and Folaron's (2003) findings that, as women engaged in prostitution, they became increasingly attached to such lifestyles. In addition, Williamson and Folaron report the social networks that women established within their prostitution-associated circles to be motivating and drawing dynamics that encouraged women to continue in their lifestyles of prostitution.

Given the numerous harmful effects of engaging in a lifestyle of prostitution, some researchers (e.g., Cimino, 2012; Sanders, 2007) have focused more efforts toward understanding the complex, complicated process of exiting prostitution. Baker, Dalla and Williamson (2010) examined four models that address cognitive and behavioral change processes, two of which specifically pertain to exiting prostitution, and proposed their own integrative model. Their model describes six stages of exiting prostitution, which draw on the strengths of the evaluated theories and seeks to correct the weaknesses: immersion, awareness, deliberate preparation, initial exit, reentry, and final exit. The formulation of these stages provides researchers with a helpful framework for understanding the significance of research on women engaged in prostitution.

The present study sought to further investigate further various dynamics that prostitution had on the lives of participating women. Previous research primarily has focused on the experiences and specific behaviors of women engaged in prostitution, so we desired to advance the research literature in this field by exploring how street-level female prostitutes came to understand the personal constructs involved with their behaviors. We believe that better understanding these dynamics, combined with previous literature regarding the motivations of prostitutes, will aid human service workers who assist this population group and can be used to inform policies and programs aimed toward helping women exit prostitution.

Since surveys fail to absorb the “thick description” (Damianakis & Woodford, 2012) or quality of information to be collected from a sample of prostitutes, we deemed qualitative methodologies to be most apt for the objectives of the present study. Participant observation approaches contained potentially unacceptable safety risks to the researchers, as well as other obvious logistical and ethical issues. Consequently, we pursued a phenomenological, qualitative paradigm as being most prudent for accomplishing the present research aims.

## METHODS

### Participants

Interviews were obtained from 25 women who were incarcerated at county jails, being arrested on charges of street-level prostitution. Ages of the women ranged from 21–42, with a median age of 29 years old. Sixteen of the participants in the sample were Caucasian, and the others were African-American. Consistent with standard qualitative research protocol, we utilized criterion sampling, selecting individuals who met the condition of interest for the aim of the present research study. Particularly, the sample represented all the incarcerated inmates (who met the standard), being located in two county jails. The prisons were located in medium-sized, Midwest cities and most participants reported growing up within a 100 mile radius or so of the jail where they were incarcerated. Due to the obvious sensitivity of the subject matter, anonymity was assured to the research participants, so we deliberately are choosing to keep demographic information about the participants to a minimum in the present article. Obviously, names used for reading clarity are pseudonyms and the study met university IRB requirements.

Saturation (Bernard, 2011) occurred during the data collection, providing reasonable assurance that the sample size was adequate for the study's objectives. Particularly, after approximately twenty interviews, we were finding that the law of diminishing returns was occurring with the data. As such, adding new individuals to the sample was not adding significant amounts of new insights to the study's overall findings. Consistent with Guest, Bunce and Johnson (2006) and Neuman (2006), we believe that the sample size was sufficient to the research objectives established for the present qualitative study.

### Procedure

Among the various types of qualitative methodology (Creswell, 2012), we designed the present investigation as a phenomenological research study. As such, our aim in the study was to obtain the perspectives of the participants and to report their perceptions, from the vantage points of their own words, ideology, and constructs (Denzin & Lincoln, 2008). One-on-one interviews occurred in conference rooms inside the jails and were tape recorded for later analysis. The interviewer was always female in order to make the interview situation as comfortable as possible for the participants, due to the sensitivity of the subject. None of the researchers have backgrounds with prostitution, so the interviewers were outsiders to the research construct (Cohen, 2000; Miller & Crabtree, 2004), affording potential greater objectivity on the part of the researchers. During data collection, we utilized semi-structured interview formats (Alvesson, 2011). This allowed the participants at times to take the interviews in diverse directions, encouraging them to share with us their own stories, cogent life impacts, and help us understand their worlds as much as they were able to do so (Potter & Hepburn, 2005). We believed that, given the complex nature of prostitution and also the other struggles these women experienced, the semi-structured format would obtain the best and most useful information for the study's objectives.

When analyzing the data, we utilized an open coding process (Maxwell, 2012). This means we approached the transcripts in an inductive manner. We did not have particular pre-conceived constructs for which we were looking. Rather, we used constant-comparison among and within the transcripts in order to identify reoccurring words, ideas, and concepts (Chenail, 2012). These generated codes that were useful in managing the analysis. Sometimes the codes were collapsed or combined, due to evident similarity in the participants' percepts. In other cases, we abandoned some codes since they lacked enough support to be representative of the sample at large (Creswell, 2008). The utilization of the qualitative analysis software NVIVO-8 helped to manage this process. However, consistent with (Lewins & Silver, 2007), we did not let the analysis process become “automated,” removing the human intuitive and subjective element out of the process. In other words, the qualitative software analysis system worked for us as researchers and not vice versa.

From the codes, themes emerged. These were constructs in the findings that were reflected in most of the participant's views (Ryan & Bernard, 2003). Consequently, all of the findings reported in the present study represent the consensus of all the participants in the study. Overall, the findings showed detrimental relational, social, and psychological effects of women in our sample engaging in prostitution activities. Due to limited publication space in the present article, we are reporting only the relational effects here.

Our intent was to generate a research study that possessed robust rigor, by qualitative research standards (Cope, 2004; De Wet & Erasmus, 2005). Internal validity for the study was enhanced in a number of ways. One was via meetings among the researchers in order to collaborate regarding potential coding strategies and thematic analysis (Bogdan & Biklen, 2007). Consequently, the results of the present study represent the results of dialogue, thorough discussion, and detailed analysis among multiple researchers who collaborated in a team effort in the present study. Additionally, we employed a qualitative researcher, independent of the data collection and analysis, to provide autonomous feedback to the researchers regarding the research questions, methodology, and analysis (Grbich, 2007). This served as a helpful, independent check on our protocol and assurance that the results we are presenting were aptly grounded in appropriate qualitative methodology and to the actual transcript data collected.

Member checking (Metro-Jaffe, 2011) was utilized in order to garner feedback from the research participants. This involves sharing the general findings with those who provided the interviews. The process allowed us to check to ensure that what we concluded in the study aptly reflected the actual sentiments of the research participants. Consistently, we found that the results presented in the present article did accurately portray what the research participants agreed were their overriding sentiments. Data trails (Rodgers, 2008) were generated in order to enhance the study's internal validity. This involved tying each of the results reported in the present article to particular quotes and citations by the respective research participants. This process has three benefits. First, it helps to ensure that each finding reported aptly represents the consensus of all the participants. Second, data trails allows other qualitative researchers to check our research for independent analysis, if desired, should anyone later suspect fraudulent research occurred. Third, data trails also can aid future researchers who wish further to explore this subject. They provide these researchers with starting points that they can use in order to enhance and further their own research designs and allow for helpful comparisons with the present one.

### Ethics

The study procedures were carried out in accordance with the Declaration of Helsinki. The Institutional Review Board of the Cedarville University approved the study. All subjects were informed about the study and all provided informed consent.

## RESULTS

Women in our study reported four psychological dynamics when relating their personal constructs about prostitution. First, participants described accounts of physical and emotional violence which they experienced at the hand of clients and others involved in the lifestyle. Next, interviewees explained an extreme dislike for their actions relating to and involving prostitution. Third, participants described how the lifestyle of prostitution had evolved into something which they viewed as a psychological addiction. However, they used the term vernacularly rather than in a medical sense. Finally, participants shared hopes they had of changing their lifestyles in the future – but also needing intervention and social service assistance to do so.

## Harmful view of prostitution

The women in our study shared many disturbing stories in which they were victims of violence and rape during the time they spent engaging in prostitution behaviors. Most participants described experiences of physical abuse, sexual assault, or both. While the details of each woman's story varied, the general theme of victimization was woven throughout the participants' lives as prostitutes. For example, Caroline recollected a time in which she was physically assaulted by a client:

*I've been hurt plenty of times. Well for instance, this scar that's on my chin right here* [pointing to her chin]. *When I asked for my money first, I was punched in the mouth by brass knuckles and forced back into the car. I had a tooth knocked out. I couldn't eat for weeks. I thought I was going to die.*

For most participants in our study, the physical violence they experienced was accompanied by sexual violence. This sexual violence may have both emotionally and physically detrimental effects. Not only must women endure the initial humiliation of being sexually assaulted, but they also may acquire sexually-transmitted diseases, sometimes without the opportunity of using protection in order to prevent this from happening. Hope, for example, discussed this risk of acquiring a sexually transmitted disease as a result of being sexually assaulted:

*Yeah, yeah it's* [prostitution] *very dangerous. Especially because there's girls out there that have HIV and still do it. Yeah, I've been like tied up and threatened to have some stuff shoved up me.*

Additionally, Crystal captured the combination of physical and sexual violence that perpetrators sometimes force on their prostitution victims:

I've been raped several, several times. I've been at gun point. Tasered. Uh, I've had a lot of guns thrown on me, held to my head. So I mean, just… yeah. Beat a lot.

Most participants described experiencing some sort of assault on multiple accounts. Among many of the women, physical and sexual assault seemed to be accepted as a part of daily life when engaging in prostitution. This acceptance phenomenon was so widespread that few women expressed feelings of self-efficacy that seemingly would encourage them to prevent future instances of physical or sexual violence.

In addition to the commonality of physical and sexual violence the women experienced, some women noted the fact that the harm they experienced while being involved in prostitution not only harmed them physically, but it resulted in emotional pain as well. Many expressed how the negative treatment of others toward them led these women to develop views of themselves portraying that they were neither good nor worthwhile people. Shannon, for example, confessed the damage she endured to her self-esteem and the shame she felt as a result:

There's some guys that act like they're police officers when you're out there and they force you. They pull knives on you and beat you up and stuff. Or, because you are soliciting and disrespecting yourself, other people, men and women, sometimes just take it upon themselves to degrade you, because you get the look about you or something. Like maybe like, sometimes, every now and then I get the strength to not use and it's like a big flashing sign around me that I'm a prostitute or something and so they'll just speak to you in disrespectful ways. They'll fight you and spit at you and stuff.

Further, participants shared awareness of the risks they were taking and the potential consequences of taking such risks. Dawn, for example, exemplified this awareness:

*It's* [prostitution] *very, very, very, very dangerous. You never know in today's world who you're getting in a car with or if you're going to get out of that car.*

Participants continually reiterated the fact that they were aware that each “trick” may be their last. However, there was little evidence that the women perceived any reasonable means of protecting themselves from future harm.

Most participants realized the imprudence of recurrently subjecting themselves to the risk of physical and sexual injury, but admitted the potential hazard was not enough to make them exit the lifestyle. The draw to the exhilaration experienced as part of the lifestyle seemed to outweigh the general concern for the women's personal safety. This caused many women to feel confused and often angry at themselves for making such poor decisions that could result in serious physical injury or death. Kathy, for example, described this incongruence between wanting to avoid harm and to continue in the lifestyle:

Because, I have been to the point where I get out of one trick's car and this is how the, I guess the sickness or the devil or whatever you want to call it or the conscience part of the person: get out, get beat, raped and everything and five minutes later get back in another car and go get high. I mean, we were talkin' the three of us girls were talking the other day about this, and it's really sad – the disease. Because we were all on our deathbed. Guy had a knife to our throat and everything. And when we get out of the one place, you get right back into another car – that's insanity.

## Disliking prostitution

Participants consistently reported disliking prostitution and the behaviors that accompany the lifestyle. The most popular reason given by women was that engaging in the acts made the women “feel dirty”. Additionally, women described how they were tired of being on the streets and having to endure the cycle of repetitively entering and exiting jail. Dawn, for example, encompassed many women's view of disdaining prostitution:

*They say this is the oldest profession in the book but I don't see how anybody – woman, dog, rat, any kind of personc – an even think about doing this* [prostitution].

When discussing their dislike for prostitution, a common theme in the accounts of the participants was the feelings of shame and humiliation that are associated with prostitution. Caroline, for example, emphasized the painful feelings she experienced after engaging in the sexual work behaviors:

*I hate them* [solicitation behaviors]*. Very much so. I feel ashamed and I don't feel clean. I feel dirty. People look at me when I'm walking down the street and people look at me; even if I'm not doing it that day, I still feel like people think that things about me. I don't like that no more.*

In addition to feeling immoral after taking part in prostitution behaviors, many participants described making an effort to distance themselves, both mentally and emotionally, from potential clients when coming into contact with them. Some women hope to prevent feeling unclean by reportedly exercising a tactic of psychologically detaching themselves during their prostitution behaviors. These women described using mild forms of psychological dissociation in order to protect themselves from the emotional pain that results from turning-a-trick. Crystal, for example, elaborated on her attempts to mentally “check-out” during a date with a client:

*No I don't enjoy it.* [I] *never have.* [I] *never have enjoyed that part* [doing tricks]*. I feel low. I feel dirty, I mean. When I trick, prostitute, however you want to put it, I'm in my own world.*

Other participants find it difficult to mentally remove themselves from the situation, and therefore employ a different tactic: remaining emotionally disconnected, evidently in order to channel their hatred toward prostitution behaviors. In this method, women described desiring desire to know as little about the client as possible. The women seemingly hope to make no affective connection with the person, so that their behaviors seem less of a reality to them. Kelly, for example, illustrated these simultaneous feelings of hatred and detachment:

*Yeah, I hate it* [prostitution]*. It sucks. I don't like anything about it. You just, you just think, speaking personally… you try not to look at someone. You don't want to know their name. You just want to do what you gotta do and go.*

## Addiction to prostitution's lifestyle

Although indicating a disdain for street-level prostitution, almost all of the participants described feeling as though they were addicted to the prostitution lifestyle. When using this term, however, they did so in a common-use of the word, not in a psychiatric sense of experience physiological or psychological dependence. Obviously, this finding seems paradoxical, since the women indicated disliking prostitution behaviors and the resultant humiliation it entailed for their lives. This seeming paradoxical principle may be true for any “addicts” who ultimately dislike the effects of addiction on themselves and their lifestyles. In this context, participants expressed feeling somewhat “hooked” on engaging in the lifestyle activities that they seemingly loathed. Two particular sub-themes emerged relating to the women's psychological addiction to prostitution's lifestyle. First, women commonly reported being involved with a fast-paced and unhealthy lifestyle. Amanda, for example, shared the sentiments of most participants in this regards:

*I hate it* [prostitution], *but I like the lifestyle. I feel like that's my family out there. I have* [a natural] *family, but I don't associate with them because the lifestyle I choose. But sometimes I hate it. I'm tired I want to go to sleep. I come to jail, I hate it but it's like I'm addicted to the lifestyle. So, people think it's just the drug use but it's not. It's an addiction to the lifestyle, too.*

Furthermore, participants cited the excitement and flexibility of the lifestyle as contributing factors to what they perceived as being a psychologically addicting lifestyle. The women reported enjoying the ability to work when and where they wanted, choosing their own clients. Additionally, participants reported having the perception that people on the street were their family, although, in reality, the women knew this percept did not square with reality. A combination of these perceived constructs led many of the women to view prostitution as both exhilarating and the rush they feel as part of their activities ultimately works against leaving the lifestyle. Jessica, for example, described the gust she routinely feels as a result of her lifestyle:

What's enjoyable? The thrill. Just, I don't know. After you do it for so long, it's like ‘Hey okay!’

However, they did not describe themselves as being sexually addicted or undergoing tolerance and withdrawal when the participants discontinue prostitution for time periods. Consequently, they did not meet the medical criteria for being addicted to prostitution in the sense of a formal psychiatric disorder.

A second sub-theme that emerged among participants regarding prostitution's psychological addicting lifestyle was that programs should be implemented specifically to treat the problem. Participants shared their beliefs that addictions to drugs (when this occurred) and the prostitution lifestyle needed separate treatment in order to aid in the effective exiting of the lifestyle. Participants indicated the necessity of such programs by sharing that, recovering from various drug addictions would not aid in their ability to overcome their perceived entrapment to the prostitution lifestyle. Kelly, for example, captured many of the women's desire to treat feeling hooked into the lifestyle:

*You know, these girls they come in here with just a slap on the hand and go. They're not going to learn that way. I don't know. They have all these, AA/ NA, those kinds of programs, stop programs. “I can get you in a program for people who come in to talk* [programs].” *I mean they have “recovering alcoholics”, “recovering addicts”, “drug addicts”, but there can't be “recovering prostitutes”? You know what I mean? Does this sound stupid to you?*

The women in our study view themselves wedged in a prostitution lifestyle from which they find it very difficult to just walk away. They know that drug addictions are challenging to overcome without special assistance and suggest that a formalized treatment program, with structured behavioral interventions such as AA or NA use, would be beneficial to them.

## Need for social services to exit prostitution

Women in this study unanimously conveyed hopes of exiting the lifestyle altogether. Participants described feeling “fed-up” with their ways of life, being exhausted, desiring simply to survive, and wanting to mend relationships with friends, family, and children. These were indicated to be reasons for desiring to change their lifestyles. Participants seemingly did not envision spending the rest of their lives on the streets and engaged in the prostitution. Rather, they aspired to depart from their current lifestyle and live as a functional unit of society. Dawn, for example, conveyed an “I'm done” attitude:

*I just hope I can say something to help somebody else. I've had many ass whoopins. I've been hit in the face with a baseball bat, my eye popped out, and I had five reconstructive surgeries… I hope I said something to help someone else. You know, I needed this. I needed to talk. You helped me; I'm done with this* [prostitution].

Additionally, most women reported having the desire to change their lifestyles, but they also were unsure whether they could do it alone or even how even to begin the process. Participants shared they did not adequately know how to exist among the rest of society, the prostitution was the predominant lifestyle they had known during their adult years. For this reason, interviewees expressed feeling unable to escape the lifestyle without outsides resources such as family and some type of formal exit program. Donna, for example, shared her great hope, but recognized her need for help from others:

Like my counselors, I feel good spirits with them, so I know they'll probably help me this time. I've been in treatments where every counselor I would get I wouldn't feel good spirits or nothing. You know, they didn't know nothing about me, and they were rude-nasty for real. But I'm going to be alright this time. I want to help people. When I get through with NOVA, I want to do stuff that keeps my mind of drugs. I need to get well first and then I should be able to do it, but I need help. I can't do it by myself.

Other participants, who also wanted to change, did not have as much hope as Donna and explained that they did not know how to accomplish such a mammoth goal as recalibrating their lives. Amanda, for example, captured the uncertainty felt by these women:

*I want to* [clean up], *It's just I don't know where to start. And I want to try, but it's hard.*

These participants were not able to picture their lives apart from prostitution, despite the fact that they seemingly possessed cogent desires to exit the lifestyle. Formal programming was indicated to be a perceived need in order to help the women in our sample make the connections needed for achieving their goals. Although social support likely existed around them in various ways, the participants expressed an explicit desire for participation in scheduled planning through social services to help them overcome what they described as besetting habits and lifestyles.

## DISCUSSION

Consistent with previous literature, our findings are congruent with Miller's (1993) finding that women involved in “street-level” prostitution face high levels of danger directly resulting from their work. In fact, participants surprisingly recounted instances of severe abuse with relatively little emotion. Temporary detachment had been implemented by our participants as regular means of coping with their daily stresses. Professionals working with women who are similar to individuals in our sample should give due consideration to those dynamics when writing treatment plans and seeking to amend participants' negative behavior patterns. That is, if women turning from prostitution hope to fully recover, then they likely will need to re-condition themselves against this seemingly ingrained tendency of emotional detachment.

As part of the article's discussion, we note that the prostitution behaviors do not meet a formal psychiatric diagnosis as an “addiction”. The purpose of the study is to provide phenomenological perspectives of the research participants, from the vantage points of their own words, constructs, and perceptions. By way of commentary on the findings, the participants did not describe themselves as being sexually addicted, in the sense of a DSM-IV-TR diagnosis. It is the overall lifestyle that they described as being “addictive,” rather than the actual sexual behaviors. When referencing the “addictive lifestyle” of prostitution, presumably, this entails a manner of enticing dress, locating potential clients, soliciting sales, generating repeat customers, and making fast money that likely is not taxed by the United States IRS. When the participants used the word “addicted”, they seemed to convey a sense of feeling “hooked” or “stuck” in their life situations. They wanted out – but struggled to achieve the freedom they desired. No dependence, tolerance, or withdrawal existed as is common with addiction from a formal medical perspective. Consequently, although we relate the word “addiction” in the present article, we do so since that is the term used by the research participants during the interviews; it is a vernacular use of addiction and not a psychiatric one. The women did not speak of being sex addicts and they generally did not enjoy engaging in the sexual acts for hire. To the research participants, being addicted to their respective lifestyles meant that they felt trapped in a situation that they found difficult to escape.

At the study's outset, we did not approach the research design to explicitly examine the matter of lifestyle addiction with the participants. As previously noted, this was an exploratory study in order to garner whatever perceptions existed and that the women were willing to relate to us. Further consideration should be given to the construct of “lifestyle addiction” as it relates to American female prostitutes. That is, since researchers possess this information, they can examine the construct specifically and in more detail, fleshing out further what we are only able to note as existing at this initial research stage.

Additionally, most women in our sample reported extensive instances of abuse. Consequently, prostitutes who wish to improve their lives and pursue recovery from all forms of addictions associated with the lifestyle will require specific aid in order to address these dynamics. For example, Burgess-Proctor (2008) found that most female victims of intimate partner violence (IPV) are hesitant to seek help, possibly resulting from similar, negative previous experiences. Burgess-Proctor also identifies help-seeking inhibitors and help-seeking promoters that affect the likelihood of women seeking needed aid when endangered, potentially associating these mechanisms with childhood experiences of violence or abuse. As women in our sample recounted their personal experiences of abuse, they admitted their foreknow-ledge of the potential danger involved with each sexual encounter. Congruent with O'Doherty (2011), intuition exercised by the women in our present study seemingly remained a cogent factor affecting women's decisions.

However, when participants discussed the addiction-feelings they experienced toward the lifestyle of prostitution itself, one dominating aspect of this “addiction” was the perceived sense of control they reported experiencing. Although paradoxical to the reality of their continual recounting of the physical and sexual abuse that results from prostitution, participants nonetheless viewed themselves as being in control of the sexual situations they encountered. Lucas (2005) sheds light on this phenomena by reporting that, not only did most women in her sample regularly engaged in sex work by choice, but they also experienced a sense of temporary psychological satisfaction resulting from prostitution activities and lifestyle variables. Such feelings likely contribute significantly to participants' feelings of addiction to prostitution lifestyles. Identifying the specific elements of sex work that cause women to feel empowered may help females who feel trapped in the lifestyle better understand their own emotional responses and feelings of addiction, as well as recovery solutions that address this particular facet of their addiction. In short, the women must learn new avenues of developing healthy senses of self-efficacy.

Another seemingly contradictory finding in the present study was that women in our sample disliked prostitution, often describing themselves as feeling “dirty” as a result of their behavior. The addiction that participants reported consequently must have had strong, if not deciding, influence on the decisions of women in our study. Work by Blecher and Herr (2005) advance this construct when they report findings that street-level prostitutes generally emphasize short-term rewards, rather than delayed gratification. Similarly, they found that motivational factors which proved to be most powerful and to be linked as the most similar to actual behavioral outcomes were those that centered on more temporary outcomes. Resultantly, as society seeks to aid women recovering from prostitution, programs should be established that specifically address this tendency. That is, the perspectives of such women who wish to change their lifestyles must transition from short-term to long-term goals and rewards if they are to see long-term recovery.

And finally, social support seems to be a cogent psychological variable that transcends our findings. The prostitutes in our study consistently described themselves as feeling disconnected from others in ways that otherwise would promote social and psychological well being. We believe, therefore, that programs designed to assist recovering prostitutes must include social as well as individual interventions. Helping them connect with healthy groups – replacing their unhealthy present social circles – is needed for successful interventions. Likewise, family therapy, in a systemic tradition (Carr, 2012), may be warranted – either in place of individual counseling or at least augmenting it. The psychological well being of these needed individuals, in part, seems tied to their needs for healthy psychological-social connections.

## LIMITATIONS AND FUTURE RESEARCH

All good research identifies and reports the limitations of a study (Price & Murnan, 2004). The sample for the present study was drawn from women currently residing in a medium-sized city in the Midwest area of the United States. Future research should seek to replicate the study in similar cities located in other regions of the country. Additionally, future researchers should conduct parallel studies in the milieu of larger cities such as New York or Los Angeles, where women may face challenges unique to larger metropolitan environments. Additionally, our sample did not include a significant number of Hispanic or Asian minorities. Conducting the study in locales where the present results can be compared with those from persons reared in wide cross-sections of minority population milieus may prove insightful and will enhance the research's external validity (Delmar, 2010).

Further, interviews with participants in our sample were conducted while women were in jail for prostitution-related activities. We suspect that time spent in jail provided participants with occasions for reflection on personal behavioral choices and resultant effects. While undergoing “reality checks” during incarceration, women may have been more prone to contemplation of life plans, such as raising their own children, than they would be at other periods during their lives. Moreover, the level of reflection, as well as content, may be qualitatively different when prostitutes are being interviewed in jail contexts compared to other interviewing contexts. Consequently, future researchers may wish to compare findings from interviews with prostitutes while in jail with responses of women while not undergoing legal penalties for their sexual behaviors.

As we noted earlier, the present study was designed to be exploratory in nature and, as such, we unearthed a research finding of women consistently noting what they conceptualized to be a “lifestyle addiction” of prostitution. Future researchers should design studies that focus specifically on this construct, having women explain their understandings of the concept, provide additional detail, and compare their behaviors against various diagnostic criteria. As such, we view the present article as reporting initial findings of what potentially could be a fertile research agenda with far more details available than what we are able to report her (given the interview data we collected).

Finally, future researchers may wish to conduct longitudinal studies where prostitutes' percepts are tracked over time. Various dynamics relating to their perspectives and behavior patterns could be more fully studied over the course of months, multiple years, or decades. Such research may obtain insight via longitudinal research designs when studying familial relationships, the effects of prostitution on women's children, as well as the long-term effects of prostitution on women's social relationships. Furthermore, most women in our sample described hopes for re-gaining custody of their children and someday raising them. Longitudinal studies would provide follow-up data regarding whether these aspirations became realties and under what conditions or contexts.
